# Understanding Older Adults’ Technology Use Preferences and Needs From a Triangular Perspective: Qualitative Study

**DOI:** 10.2196/72716

**Published:** 2025-11-11

**Authors:** Ortal Cohen Elimelech, Sara Rosenblum, Michal Tsadok-Cohen, Simona Ferrante, Naor Demeter

**Affiliations:** 1 Department of Occupational Therapy The Laboratory of Complex Human Activity and Participation University of Haifa Haifa Israel; 2 Department of Electronics, Information and Bioengineering Politecnico di Milano Milan Italy; 3 Department of Occupational Therapy Faculty of Social Welfare and Health Sciences University of Haifa Haifa Israel

**Keywords:** daily activity, matching person and technology model, qualitative study, older adults, COVID-19

## Abstract

**Background:**

Technology use is increasingly integrated into daily life, including among older adults, whose adoption and engagement with technology warrant closer examination. According to the matching person and technology model, technology adoption is more likely when a person’s preferences and needs align with a device’s functions and features, as well as the environment in which it is used. However, factors such as age-related changes, evolving preferences, and the rapid pace of digital transformation complicate this process. Additionally, older adults often rely on support from family members and health professionals, yet their perspectives remain largely unexplored.

**Objective:**

This study aimed to examine the daily technology use of older adults in Israel from a triangle perspective, incorporating the views of older adults, family members, and health professionals. It explored preferred technology-based activities, device features, the shift from in-person to technology-based interactions, and responses to mismatches between preferences, device characteristics, and social support.

**Methods:**

Nine web-based focus groups, each with 6 to 7 participants, were conducted during the COVID-19 pandemic (February 2021 to July 2022). Participants included 20 healthy, independent older adults (10 women, aged 66-80 years), 19 family members (children and grandchildren, aged 21-55 years), and 20 health professionals with at least 5 years of experience working with older adults.

**Results:**

Older adults demonstrated diverse preferences for technology use in daily activities, shaped by urgency, significance, and device characteristics. This perspective was reinforced by family members and health professionals, who highlighted the importance of distinguishing between technology types based on their users and features. Additionally, older adults expressed mixed views on shifting from in-person interactions to digital alternatives, while family members and health professionals emphasized the importance of social interaction for well-being. Finally, older adults described significant emotional challenges when navigating technology. Family members and health professionals identified key contributors, including the gap between perceived and actual technological abilities, generational differences in digital exposure, and cognitive demands associated with technology use.

**Conclusions:**

This study highlights the significant variability in older adults’ daily activity preferences, which strongly influences their technology use. It suggests shifting the focus from technology to its practical application in meeting individual needs. In this context, it is important to consider the need for social interactions. Addressing social interaction needs and emotional challenges is crucial, as unmet technological needs can lead to frustration and disengagement. These insights can inform strategies to enhance technology use among older adults by aligning technology design and support systems with their preferences and needs.

## Introduction

### Background

Globally, fundamental demographic change has reshaped the face of society: the population is aging. According to the World Health Organization [1], the proportion of adults aged 60 years and older is projected to double, rising from 12% to 22% by 2050. This change presents significant challenges, requiring the development of effective strategies to promote physical and mental health [2]. Integrating everyday technology—computers, mobile phones, and tablets—holds considerable promise for improving older adults’ health and engagement in meaningful activities [3]. These daily technologies can support a range of practical and financial tasks (eg, information searches, product comparisons, and banking) and social and leisure activities (eg, gaming and social networking) [4].

Daily technology use becomes increasingly important when older adults experience health threats and when routine activities are affected. These situations range from extreme weather events to public health emergencies like the COVID-19 pandemic. In the context of the pandemic—a time of restrictions on daily activities—technology played a pivotal role in enabling older adults to adapt their routines, sustain meaningful engagement, and maintain their health [5]. As expected, older adults increased their technology use while desiring tools better tailored to their preferences and needs [6]. Therefore, with the rapid growth of the global aging population, it is essential to understand and address older adults’ preferences and needs to better align daily technology with their expectations and requirements.

The matching person and technology (MPT) model, developed by Scherer, emphasized the importance of optimizing the alignment between users and technology. Although designed for assistive technology, it can also be applied to daily technological use [7]. This model identifies 3 key factors that distinguish technology users: the user’s preferences and needs, the device’s functions and features, and the environment in which the technology is used [8]. When technology fails to meet an individual’s preferences or lacks adequate environmental support, users may avoid using the technology or experience significant frustration. Conversely, a strong person-technology match can be achieved when a device aligns with user expectations and is perceived as beneficial, enhancing technology acceptance and use [9]. By integrating the MPT framework, this study aims to understand the model’s concepts and their interrelationships. Specifically, the study examines the experiences of older adults in Israel with daily technology use based on their reports and those of their social environment, including family members and health professionals.

When analyzing older adults’ preferences and needs regarding daily technology use, 4 primary challenges complicate a comprehensive understanding of these preferences and needs [6]. The first challenge stems from age-related declines in sensory, motor, and cognitive abilities, which are essential for effective technology use [10,11]. Sensory and motor abilities play a critical role in engaging in conversations, hearing communication partners, reading text, and interacting with touchscreens. Similarly, declines in various cognitive abilities—such as memory, attention, and problem-solving—can hinder older adults’ capacity to process large amounts of information and address technical issues. As a result, older adults often require a slower learning pace when adapting to new technologies, as their age-related needs demand additional time and tailored support compared with younger users [12,13].

The second challenge arises from the persistent mismatch between older adults’ unique age-related needs and how technology is designed [6]. Technological development frequently relies on assumptions made by developers about older adults’ requirements, leading to designs that fail to address their specific challenges adequately [14]. For example, a device’s functions and features, such as small text sizes, complex operating systems, or extremely fast interfaces, may not accommodate age-related sensory, motor, or cognitive changes [3].

In addition to these design shortcomings, addressing older adults’ technological needs becomes even more complex, given the wide variability in age-related changes. Since the aging process is experienced uniquely by each individual [1], personalized adaptation represents a third significant challenge. Older adults exhibit diverse interests and preferences for technology use in their daily lives, spanning activities such as leisure, social interaction, physical health management, and education [3,15]. These preferences are highly dynamic, further complicating the development of technologies that effectively address their user needs [3,15].

Van Boekel et al [4] highlighted this variability by classifying older adults into 4 groups based on internet usage preferences. The first group consists of individuals who primarily use the internet for practical and financial tasks, such as comparing products or web-based banking. The second group uses the internet minimally, focusing mainly on email and basic information searches. The third group adopts a broader range of activities, including reading news, video calling, and watching television. Last, the fourth group primarily uses technology for social and leisure activities, such as playing games and interacting on social media. Additionally, qualitative research on community-dwelling older adults highlights the importance they place on maintaining independence in daily activities, with technological device characteristics—such as size, weight, and usability—playing a critical role in adoption decisions [16]. This variability in daily technology use highlights the inherent complexity of designing and tailoring technologies to meet older adults’ diverse, evolving preferences and needs.

The complexity of older adults’ diverse preferences and needs is further compounded by the digital transformation of daily activities, which represents the fourth challenge. The digital transformation has fundamentally altered social engagement patterns among older adults, particularly by migrating traditional interactions to web-based platforms [17]. The widespread transition of routine activities—such as banking—from in-person interactions to digital management signifies a major shift in social connectivity. Despite the increasing prevalence of digital platforms, face-to-face interactions remain essential for maintaining older adults’ health and social well-being [17,18]. This technological transition can significantly influence older adults’ attitudes toward technology adoption as well as their preferences and needs [19].

However, a significant research gap exists regarding how older adults and people in their social environments perceive and interpret the technological transition of daily interactions. Similarly, while existing findings on preferences and needs for daily activities are valuable, they predominantly reflect older adults’ perspectives, neglecting the views of others in their social environment—such as family members and health professionals—who are generally familiar with technology, and often play critical roles in supporting older adults’ daily technology use challenges [6,16,20].

Specifically, integrating the social environment perspective of older adults in Israel is crucial, as family members and health professionals are deeply involved in older adults’ daily lives and can offer unique insights into how technology might better align with their preferences and needs [21]. Israeli society is characterized by collectivist and family-oriented values [22] and therefore offers valuable insight into the role a specific sociocultural context plays in shaping older adults’ technology preferences. Cross-cultural research, conducted during the COVID-19 pandemic, has highlighted such differences, showing that Italian participants prioritize technology for physical activities, Mexican participants for family communication, Portuguese participants for accessing knowledge, and Spanish participants for spiritual and health support [23]. As qualitative research aims to provide deep, contextual understanding rather than broad generalizability, the focus on the Israeli context, with its distinctive family-oriented values, offers valuable insights into how cultural factors shape technology adoption.

Although older adults frequently require support in navigating daily technology challenges, the support provided by people in their social environment may not consistently align with their unique abilities and needs, such as accommodating a slower technology learning process. This situation can potentially intensify emotional responses [24]. Older adults navigating digital transformation can frequently encounter challenges extending beyond practical difficulties to encompass profound emotional dimensions [12,13]. These challenges manifest in diverse forms, including pervasive anxiety [25], persistent feelings of inadequacy [26], and deep concerns regarding the possibility of technological errors [27]. Such emotional responses, often shaped by the intricacies of daily technology use, can substantially influence technology adoption and continued usage. However, existing research predominantly concentrates on initial technological responses, overlooking the nuanced emotional dynamics associated with ongoing technology use. This research gap severely limits our capacity to develop targeted emotional support strategies that genuinely resonate with older adults’ technological needs and preferences [28].

### Research Aim

Considering the gaps in the literature and integrating the principles of the MPT model, this study aims to understand the experiences of Israeli community-dwelling older adults regarding their daily use of technology from a 3D perspective: older adults, family members, and health professionals. The study seeks to understand (1) the daily activities older adults prefer to conduct and the associated functions and features of technological devices, (2) the experiences of older adults and their social environment following the transition from in-person interactions to technology-based interactions, and (3) the emotional responses of older adults to mismatches between their preferences and needs as users, device features, and environmental support, as well as the factors influencing these responses. Given that this study was conducted during the COVID-19 pandemic, the context of daily technology use was the home environment.

## Methods

### Overview

This study was part of the larger project “Empathic Platform to Personally Monitor, Stimulate, Enrich, and Assist Elders and Children in Their Environment (ESSENCE),” which used opportunities during the COVID-19 pandemic to examine vulnerable populations, including older adults and children. The focus groups addressed broad questions about daily function, health, and technology use during the COVID-19 pandemic. This study reports on focus group findings regarding the daily technology experiences of older adults, incorporating the perspectives of family members and health professionals. The focus groups provided a multiperspective lens, offering valuable insights into participants’ preferences, needs, and the functions and features of technological devices concerning their daily technological use. This study adheres to the Consolidated Criteria for Reporting Qualitative Research guidelines [29] (Multimedia Appendix 1).

### Procedures

#### Data Collected

Data were collected between February 2021 and July 2022, during which parts of Israel were in lockdown. Data collection continued until the main issues were consistently repeated and theoretical saturation was reached [30].

The study incorporated 3 distinct types of focus groups—healthy older adults, family members, and health professionals—allowing for triangulation and enhancing the validity of the findings [31]. Participants were recruited using snowball sampling and social media platforms. Interested participants (older adults: n=34; family members: n=26; health professionals: n=24) contacted the researchers via email and received a detailed explanation of the study. Potential participants were then screened for eligibility. Eligibility criteria for older adults required fluency in Hebrew, living independently as reported by the participants, and adequate technical ability to operate Zoom. Eligible family members were required to be Hebrew speakers who maintained regular weekly (or more frequent) contact with their older adult relatives. Health professionals were also expected to be fluent in Hebrew and have at least 5 years of experience working with older adults. Upon receiving information about the study participants who met the eligibility criteria and were available to participate, they completed a web-based consent form and a demographic questionnaire.

#### Participants

##### Overview

A total of 9 focus group sessions were conducted, with group sizes ranging from 6 to 7 participants, as recommended by Krueger and Casey [32].

The focus groups were categorized into 3 clusters.

##### Older Adults

Twenty older adults (n=20), with a mean age of 70.8 (SD 3.81) years, participated in the study. All lived independently and reported proficiency in using a computer and Zoom software. Half of the participants were female (10/20, 50%), and the average number of years of education was 15.7 (SD 2.45).

##### Family Members

Nineteen family members (n=19) familiar with the daily routines of older adults participated. The majority were female (16/19, 84%), with a mean age of 39.16 (SD 10.52) years and an average of 16.5 (SD 3.29) years of education. Family ties were categorized into first-degree (15/19, 79%) and second-degree (4/19, 21%).

##### Health Professionals

Twenty health professionals (n=20), including occupational therapists (6/20, 30%), nurses (4/20, 20%), physiotherapists (3/20, 15%), social workers (3/20, 15%), doctors (2/20, 10%), and day center directors (2/20, 10%), participated in the study. Their mean age was 49.85 (SD 11.75) years, and the majority were female (18/20, 90%). On average, they had 17.95 (SD 9.49) years of experience working with older adults.

#### Research Tools

The first and third authors conducted web-based focus groups with 2 women who are also occupational therapists with a strong personal and professional interest in the research topic. Acknowledging that some participants in the health professional groups had preexisting professional relationships, the moderators created a relaxed and open environment to ensure inclusive and balanced interaction. Additionally, due to the snowball sampling method, a few participants had prior acquaintance with one of the moderators. To mitigate any potential influence from this, each focus group was co-facilitated, ensuring the presence of a second, neutral moderator to maintain a balanced discussion. While the moderator’s questions were tailored to each focus group, the session structure was consistent across all 3 groups (Multimedia Appendix 2). The complete interviewers’ guide has been published in a related study [33]. This structure, developed based on the relevant literature [34], was designed to create a comfortable environment where participants felt free to share their thoughts and experiences. The session began with an introductory question, “What is your most enjoyable activity in your free time?” to promote engagement. Follow-up questions explored older adults’ daily technology use, including the following:

- In your daily life, how do you typically use technology?
- What types of devices (like smartphones, computers, tablets) do you use, and what do you typically do with them?
- When you use technology, what aspects do you find most challenging or difficult to manage?
- What are some things you wish you could do with technology but are currently unable to?

#### Data Analysis

One focus group session was conducted separately with each participant group. Each focus group session lasted approximately 60 to 70 minutes and was conducted via Zoom videoconferencing. The sessions were video- and audio-recorded in Hebrew.

Researchers recorded reflective comments capturing verbal and nonverbal interactions during each session. Given the web-based nature of the sessions, researchers paid particular attention to the technological needs of older participants, including support with internet setup and technical difficulties. These efforts contributed to the study’s credibility by ensuring a broad perspective on the older adults’ use of technology.

Data were analyzed separately for each group using thematic analysis and the constant comparative method [35,36]. The transcripts and reflective notes were reviewed repeatedly to familiarize the researchers with the data. Memos were created using Microsoft Word and Excel to assist with data organization and categorization. The data were sorted, coded, and compared across different cases. The first and last authors independently coded the data. Initial comprehensive coding identified overarching themes, followed by selective coding (a total of 48 codes were identified) to refine the categories. Quotations were then rearranged into new categories and translated into English. Researchers focused on the similarities and differences in participants’ experiences across the 3 focus groups. Considering that the study’s themes emerged from a dynamic across older adults, family members, and health professionals, individual participant checks were less relevant to capture the complexity of the findings. Furthermore, in-depth discussions among the researchers, including a qualitative researcher and a field expert, were held to resolve disagreements and enhance the findings’ trustworthiness [34].

### Ethical Considerations

The study protocol was reviewed and approved by the University of Haifa’s Faculty Ethics Committee (approval number 086/21). After receiving information about the study aim and procedure, participants provided web-based informed consent before participating in the study and were advised of their right to withdraw at any time. To ensure confidentiality, focus group sessions were audio- and video-recorded, transcribed, and anonymized by removing all identifying details. The original recordings were stored on password-protected computers and were permanently deleted after transcription. To protect participant anonymity, all names appearing in the Results section are pseudonyms, and some of the background information was changed (eg, place of living). In addition, participants were asked to maintain confidentiality about other participants’ contributions. Participants received compensation in appreciation of their time. Older adults and family members received US $60, and health professionals participating received US $268. This conversion rate reflects the approximate value at the time the study was conducted. Compensation rates differed according to the participants’ positions; health professionals were compensated for their professional time and expertise, while other participants were compensated for their appreciation for sharing their experiences.

## Results

### Overview

Based on the analysis, 3 main themes emerged. (1) Meaning-driven choices highlight that older adults show varying preferences for daily activities involving technology, shaped partly by the activities’ significance and urgency and the devices’ characteristics. Family members and health professionals reinforce this perspective, emphasizing the importance of distinguishing technology types based on their functions and device features. (2) Changing social landscapes reflect older adults’ diverse views on the transition from in-person to technology-based interactions. Although some perceived technology as a valuable tool for saving time and money, others described it as impersonal and lacking human connection. Family members and health professionals underscored the vital role that social interactions play in the lives of older adults. (3) Emotionally related technology challenges emerged prominently throughout all groups, even though no questions directly addressed the emotional experience of using daily technology. Older adults described their emotional experiences when faced with technology challenges. Family members and health professionals explored the reasons behind these emotional reactions. This applies to the gap between how individuals perceive their ability to use technology and how they actually use it, the fact that this generation was not born into a technological environment, and the cognitive abilities required to navigate technology.

### Meaning-Driven Choices

The findings reveal that older adults’ engagement with technology is not uniform; instead, it is driven by a diverse set of individual needs, goals, and preferences. For example, Luna, a 75-year-old woman, explained that she learned to use technology to accomplish her professional goals:

There are many things on the computer that I’m not proficient enough in, not utilizing enough. But I don’t have anything urgent because what was urgent for me already, for example when I had these lessons, it was urgent for me to learn how to conduct meetings on Zoom, so I’ve already learned that.

Luna’s repeated use of the word “urgent” suggests she valued activities that led her to become familiar with technology. She elaborates on her technological engagement and describes various practical applications: “It depends on your needs. I also use banking services and weather services, etcetera.” Similarly, Dylan, a 72-year-old man, described how his passion for traveling motivated his proficiency with technology: “I needed technology outdoors. Since I’m a traveler, I love maps - all kinds of navigation tools. Topographic maps, Jeeps. I’ve made a lot of progress [with technology use].” Beyond his travel-related technology use, Dylan detailed how digital platforms facilitated social connections during the COVID-19 pandemic: “During all these periods, there are of course Zooms, and all kinds of lectures, even WhatsApp chats between groups of friends - from elementary school and from the army.”

Luna and Dylan provided examples of how their interests drove their engagement with technology. This highlights the distinctly individualized and nonuniform nature of digital engagement among older adults. Older adults also displayed many preferences regarding devices. Some prefer to use a smartphone, like Lydia, a 74-year-old woman, described:

More on the phone, because it’s mobile, and that’s why I also asked you to send me the link to the phone, to this small device, and not to the computer. Because it’s more convenient, you can sit wherever you’re sitting and continue to communicate, and you don’t need to sit in front of the computer, in front of the device itself.

Nevertheless, some older adults preferred computer use, as Ethan, an 80-year-old man, explained:

For me, it’s actually the opposite. I try to use the computer as much as possible and not the phone. I do have apps, but I prefer the computer. All my interactions with the bank and all the online shopping I do–and I do quite a lot–I only do through the computer.

Lydia and Ethan demonstrated different preferences for the device based on its characteristics, including its size and usability in various settings. Consequently, Maeve, the daughter of a 69-year-old woman, highlighted the differing computer usage needs between her parents based on their different daily activities:

Basically, most of my dad’s work involves computers. She (my mother) has her own computer, but it’s more for leisure than work. My mom spends a lot of time on the computer, mostly email, games, and movies. Also, she prints things for some women’s group, another kind of volunteering.

According to Maeve, her father uses the computer primarily for work activities. In contrast, her mother uses it more for leisure activities, illustrating how the same technology may be used for different purposes depending on needs and preferences. Health professionals extended this idea, suggesting that differentiating between the functions of technology is possible, as a daycare manager stated:

I think distinctions should be made between using technology for functional uses—that is, health insurance, shopping—versus alleviating loneliness and games.

### Changing Social Landscapes

Older adults expressed a range of perspectives regarding social interactions, spanning from in-person to technology-based formats. In some cases, this change was viewed positively, emphasizing its benefits to their lives. For instance, Zev, a 69-year-old man, reflected on this transition, stating:

Why do we need to go there [bank services]? To waste our time in lines? I’ll do everything I need through the computer—payments or this or that. And that’s where my process with such things ends. Why go and stand in line? To search for parking? All those troubles have spared us.

Zev also described his experience with the visa application process: “I got a US visa in five minutes while sitting in front of the computer.” Similarly, Michael, a 70-year-old man, explained the benefits of technology:

It has many more advantages than disadvantages. We have a daughter in the United States right now, so we are with the granddaughters—they have two daughters—and we talk with the family almost every day. It’s both free and brings us closer together. It’s very accessible and user-friendly, and we just have to say thank you to all the apps and technology that are doing us so much good.

Zev and Michael highlighted the benefits of using technology, including saving time, lowering costs, and being accessible. However, other older adults expressed concerns about the shift to remote formats, highlighting a significant drawback—the loss of human interaction. Bianca, a 73-year-old woman, expressed a different viewpoint from Zev:

In the past, you would go, and there [bank services] was a personal touch. “Hello, Mrs… how are you? How can I help you?” Today, it’s done over the phone. But that’s okay if they know… They’ve also learned that they need to be much more polite, much more communicative.

Bianca emphasized technology’s more impersonal nature and preferred human contact. Likewise, Lydia, a 74-year-old woman, described technology’s inhuman characteristics:

Technology is also something cold and inhuman, as you know, so I always prefer personal contact whether it’s going to the health clinic or calling to make an appointment by phone. With the internet, it’s very… I don’t like it; I haven’t connected with it.

According to Lydia, using technology prevents her from communicating with people, including routine activities such as health care, emphasizing the importance of social interaction in her life. Considering the context of COVID-19, older adults experienced using technology for activities they used to carry out face-to-face. Oliver, a 69-year-old man, articulated his preference for in-person activities as follows:

I signed up to learn Arabic on Zoom and I saw that it didn’t really suit me. I prefer face-to-face. So, after five or six lessons, I stopped. So, these are the kind of things we’re waiting for the coronavirus to end so we can meet in person.

The social aspect of Arabic lessons, which cannot be achieved using technology, plays a crucial role in Oliver’s engagement in such activities.

Family members provided their perspectives based on their own experiences, highlighting the importance of social interaction for older adults. For instance, Adina, the granddaughter of an 85-year-old woman, explained:

We have a group with the whole family, including uncles, cousins, and all that. She is active, she reads, and she sees everything, but it’s still not as natural for her as it is for us. So, while we can maintain contact with distant friends through technology and consider it staying in touch, for her it’s less so. It fulfills a different role in her life.

Despite Adina’s granddaughter engaging in web-based interaction with her family, it seemed to have a different meaning for Adina and did not fully meet her need for social interaction.

Health professionals emphasized the importance of face-to-face social interaction for older adults. Hannah, a family therapist, shared an illustrative example from her support group:

In the support group, I have someone who is in really good shape, but she doesn’t do two things in the same day. She doesn’t go shopping, to the clinic, or to the post office all in one day so that she has a reason to leave the house. She said, “I see the clinic, but I won’t go in so that I have something to do tomorrow.” And she won’t go to the post office on the same day either. She’s fully capable of using technology, but she wants the outing.

This example illustrates that despite the ability to use technology, outdoor activities that include in-person social interaction remain an important part of older people’s lives. It highlights how some older adults deliberately structure their routines to ensure regular outings and face-to-face social encounters, even when technology could potentially replace these activities.

### Emotionally Related Technology Challenges

Even when older adults use technology for daily activities, they often face challenges, resulting in embarrassment, frustration, fear, and anxiety. Ariel, a 75-year-old man, described this feeling as relating to the device’s characteristics:

What isn’t annoying? A touchscreen that’s less responsive, a smaller screen, and seeing the whole world almost the size of a postage stamp—it’s just not convenient.

Ariel became frustrated by a device that appeared unsuitable for his abilities. Nila, a 73-year-old female, described her challenges logging into the Zoom focus group:

The example was today when I tried to expose myself to Zoom, and I did it before… I found myself quite at a loss… I felt a bit foolish. I said wow, I’m trying for a quarter of an hour to open it, and it opens for me, but I don’t know how they will see me and how I will see.

Nila expected to connect to Zoom as she had done previously. However, she felt lost when encountering technical difficulties. Likewise, Naya, a 66-year-old woman, explained the challenges she faced trying to resolve navigational problems while driving:

I don’t know how to fix situations, like if Waze suddenly disappears or something like that, then I need… then I get lost there. I try to tap here, there, it doesn’t work for me. These are things that if my husband is next to me… then he immediately fixes it. That’s the problem, I rely on him.

Naya’s experience of correcting the situation and her need for assistance caused her to feel lost and reliant on others to use the technology effectively.

Similarly, family members provided perspectives on the emotional reactions the older relatives experienced when they encountered technological challenges. Shela, the daughter of a 66-year-old woman, provided an insight into the experiences of her mother with her new smartphone:

She bought an upgraded phone, and it drove her crazy. She couldn’t set the ringtone, and she didn’t ask for help either… So, I called her and said, “But we can’t hear your ringtone,” and she replied, “Yeah, I couldn’t set it up.” It really makes her feel small, like she even says, “How stupid am I?” Not stupid, but words like that or she apologizes a lot, “I should have known this”.

Shela’s mother is eager to become proficient with technology, yet still encounters barriers that negatively impact her sense of self-worth regarding her ability to use it. Such an emotional reaction can be the result of the gap between how Shela’s mother perceives her ability to use technology and how she actually uses it.

Nancy, the daughter of an 80-year-old man, shared her experiences with technological customer services:

When I call customer service for… my TV or my mobile phone, and they start explaining, I have no idea what they’re talking about. It’s like I would say to my kids, “Here, take it, I’m not dealing with this.” Like, and I think that even the company employees working there, they don’t understand that I don’t understand. Now if they’re talking to my father, they understand even less than he doesn’t understand, and it’s terribly frustrating for him and it’s terribly frustrating for them.

Nancy explained how customer service interactions often use language with technological terms unsuitable for her or her father’s level of understanding. This mismatch in communication leads to frustration for the older adults and the service representatives. Nancy further explained how constant technological changes, coupled with the decreased adaptability that comes with aging, can lead to frustration and distress:

I think it’s a problem because it’s [technology] constantly changing. As we get older, it becomes harder for us to adjust, harder for us to adapt. I see this myself too. When something on the computer suddenly changes or there’s a version update and something suddenly doesn’t work for me, I go into a drama. So, if I go into a drama, then I think about older adults whose thinking is even more rigid and more structured and even less flexible, so it’s complex.

Health professionals provided additional reasons for such emotional reactions because this generation is unfamiliar with technology. Abigail, a nurse, addressed this issue as follows:

I think it’s a fear of the unknown. It took my mother years and years just to agree to have a mobile phone. It wasn’t even an option—I’m either at home, at work, or on my way in between, meaning, there was no understanding of why she needed it at all. And by the time she did, adding even more new technology and calls is just overwhelming. It’s all out of fear. We managed without it all those years, so suddenly the world has changed for them. They’re living in such drastically different times in terms of technology, and it’s really intimidating.

Abigail gave an example of her mother, illustrating how older adults who are not accustomed to technology may continue to conduct their daily activities without it. This can prevent them from understanding why they need it and often leads to feelings of fear. Thus, insufficient knowledge can trigger emotional reactions. Alma, a gerontologist, shared:

There’s a certain hesitation and fear of technology, that maybe it will constantly film me, and it’s kind of like Big Brother… and it will record. We’ve seen people who want to join the program [a program that connects older adults online to various activities, such as receiving medical care] but because of this thing, they simply refuse. And I can understand that because technology isn’t always fully understood by them.

In addition, Adi, an occupational therapist, discussed the cognitive abilities required for using technology and the emotional reactions it may cause:

I think beyond the learning aspect, the actual handling of the phone—which we don’t even think about—there’s an element of problem-solving. Solving problems needs to be strong and it’s how we deal with them. For example, if I accidentally exit the screen, I can figure out how to get back in a second. When they exit the screen, they are lost, and they don’t know what to do next.

Adi illustrated how problem-solving skills are necessary to navigate technology. When older adults encounter unfamiliar situations with devices and lack troubleshooting ability, they may feel lost, as Naya described above in relation to her experience with Waze.

## Discussion

### Principal Results

This focus group study examined older adults’ technology use from 3 perspectives, offering a comprehensive understanding of the factors influencing their engagement with technology. Following the MPT model, this study focused on older adults’ preferences and needs, the device’s functions and features, and the mismatch between them [8]. Older adults displayed diverse preferences for technology use in daily activities, shaped by the urgency and significance of the activity and the device’s specific characteristics. Regarding social interactions, older adults expressed mixed views about transitioning from in-person interactions to digital alternatives. Family members and health professionals emphasized the critical role of social engagement in promoting older adults’ well-being, highlighting the importance of balancing technology use with face-to-face socialization. Furthermore, older adults reported substantial emotional challenges when navigating technology. Family members and health professionals identified several key factors contributing to these challenges, shedding light on older adults’ emotional challenges in their interaction with technology.

The findings should be interpreted considering the COVID-19 pandemic, which occurred at the time the data were collected. During this period, significant restrictions were placed on daily activities, making technology a crucial tool for older adults to remain in contact with family and friends and maintain essential routines [5,6]. Accordingly, the pandemic drove older adults’ increased reliance on technology, shaping their preferences and needs, with their homes being their primary use environment. However, the findings could be relevant in understanding how older adults use and adapt technology under various challenging circumstances that affect their daily routines and social interactions.

### Older Adults’ Preferences and Needs for Daily Technology

Consistent with previous research [37], this study underscores the variability in older adults’ preferences and needs regarding technology use in daily activities. Participants, including older adults, family members, and health professionals, described many technology-related activities, including work-related tasks, leisure activities (eg, traveling, gaming, watching movies), financial management, and online shopping. Preferences for these activities differ across individuals and fluctuate within the same individual depending on the purpose, context of use, and the specific features and functionalities of the device [38]. These findings suggest that integrating technology into daily activities is pivotal in shaping older adults’ evaluation, adoption, and sustained engagement with technological tools. This study underscores how older adults’ preferences for technology use—driven by the nature of their daily activities—are key to fostering their interaction with and acceptance of technology.

The findings underscore older adults’ preferences and needs for meaningful social interactions. In some cases, older adults perceived technological changes positively, listing benefits such as saving time, avoiding parking searches, completing web-based forms, and maintaining contact with distant relatives. However, in other cases, older adults described losing interactions with others as a significant disadvantage. Family members and health professionals also emphasized this concern, highlighting the importance of face-to-face interactions.

Although previous research suggested that technology can enhance social interaction [39], this study indicates that older adults often perceive technology as cold and impersonal. This perception was evident in their strong preference for face-to-face interactions, particularly during the COVID-19 pandemic. According to the research conducted during the COVID-19 period, older adults’ contact with others in person has significantly declined [40], possibly intensifying their desire for meaningful human interaction. However, the concerns that technology may replace human interaction are not a new phenomenon and have been raised for more than a decade [41]. This indicates that the necessity of human connection is a major priority for older adults, not just a consequence of the pandemic. Therefore, rather than solely considering the functionality of daily technology use, it is also crucial to consider social elements and ensure that technology adoption aligns with older adults’ preferences and needs for in-person engagement.

The theoretical implications of these findings align with the Unified Theory of Acceptance and Use of Technology (UTAUT), which identifies behavioral intention as a key determinant of technology adoption [42]. A central element of the UTAUT model is performance expectancy, defined as the extent to which individuals believe technology will enhance their ability to perform specific tasks [43]. Another central element of the UTAUT model is perceived usefulness, which refers to how an individual believes technology will enhance specific activities. The findings suggest that although older adults recognize the functional benefits of technology, their intention to adopt it is influenced more by its alignment with their broader social needs, particularly their preference for meaningful interactions. Combining the MPT model, which emphasizes the alignment between user preferences and device features, a key practical implication involves providing a clear explanation of how certain technologies address older adults’ social needs, and thus encouraging their adoption.

### Device Functions and Features

Older adults’ preferences were evident in their device functions and feature choices. Preferences varied based on device characteristics, such as size and usability in diverse settings. Because there are plenty of opportunities for daily activities on each device, it is important to understand the individual’s needs. Although older adults represent a heterogeneous group related to their aging process, their needs may relate to their cognitive, sensory, and motor abilities [3]. For example, an individual with cognitive difficulties might prefer a simpler device, while someone with a higher cognitive ability may prefer a more complex one [44].

Family members and health professionals extended this perspective, suggesting that devices could be distinguished based on their use rather than on the device. As one device can be used for multiple purposes (eg, managing health insurance, playing games), this approach better emphasizes technology’s practical application in meeting individual needs. On a practical level, technology design can leverage the Principles of Universal Design [45]. Following this framework, devices intended for essential activities, such as managing health insurance, should prioritize simplicity and intuitive operation. In contrast, those designed for entertainment purposes, such as games, could offer greater flexibility, allowing users to customize the experience according to their personal choices and abilities [46].

### Emotional Response: Person Technology Match

This study explored older adults’ experiences when a mismatch occurs between the individual and the technology, leading to various challenges. Consistent with prior research [47], older adults identified these challenges as arising from device functions and features that fail to meet their needs (eg, small screens) and the complexity of certain tasks (eg, an application that unexpectedly disappears). Family members and health professionals emphasized that insufficient knowledge about technology and cognitive challenges significantly contributes to these emotional responses.

Thus, the findings highlight the relationship between individual abilities (eg, sensory, motor, and cognitive) and the emotional responses associated with technology use [48]. Despite their challenges, older adults continue to engage with technology, motivated by its benefits at the activity level. These benefits, which may include feelings of youthfulness and the promotion of healthy aging [44], underscore the importance of addressing the gap between older adults and technology while considering the emotional responses [49].

### Limitations and Future Research

There are several limitations associated with this study. First, the focus group was conducted via web, which required participants to have a certain level of technological proficiency. Consequently, individuals who are less proficient in the use of technology were underrepresented in the sample, thereby limiting the generalizability of the findings to the entire older adult population. Despite this, the study still revealed variability in preferences and needs regarding daily technology use. Second, the data was collected during the COVID-19 pandemic, which may have influenced older adults’ preferences and needs due to the unique circumstances of this period.

In addition, family members and health professionals who participated in the study were exclusively female, potentially leading to gender bias and limiting the diversity of perspectives. Previous research has highlighted gender differences in the intention to adopt and use technology, both within the general population and specifically among caregivers of people with dementia [50]. However, it is important to consider this finding within the Israeli context, where informal and professional caregiving roles are predominantly filled by women [51].

Last, the study’s sample was confined to Hebrew-speaking older adults in Israel, which restricts the generalizability of the findings, as preferences for technology are known to be significantly shaped by sociocultural context [6]. Future studies should address these limitations by including participants with varying levels of technological proficiency, conducting research during periods with fewer restrictions on daily activities, and ensuring a more balanced representation of genders and cultures to provide a broader range of insights.

### Conclusions

This study highlights that preference variability for daily activities is crucial in shaping the technology usage of older adults in Israel in their everyday lives. Moreover, it is essential to account for the social dimension, ensuring that technological adoption aligns with older adults’ preferences and their need for meaningful in-person engagement. The findings further suggest that distinguishing devices based on their uses emphasizes their practical applications, shifting the focus from the technology itself to how it meets individual needs. Addressing the emotional responses associated with technological challenges is critical for promoting successful technology use and integration. As our world becomes increasingly digital, understanding and responding to these diverse preferences and needs becomes paramount for ensuring that technological advancement truly enhances, rather than diminishes, the quality of life for older adults. Indeed, tailoring technology in this way may promote daily engagement, which, in turn, may improve older adults’ physical and mental health [5].

## Appendix

Multimedia Appendix 1 COREQ checklist.

Multimedia Appendix 2 Focus group interview guide.

## Abbreviations

ESSENCE: Empathic Platform to Personally Monitor, Stimulate, Enrich, and Assist Elders and Children in Their Environment
MPT: matching person and technology
UTAUT: Unified Theory of Acceptance and Use of Technology

## Data Availability

The datasets generated or analyzed during this study are not publicly available due to protecting participant confidentiality, but are available from the corresponding author on reasonable request.
