# Supplementary material for: Understanding Older Adults’ Technology Use Preferences and Needs From a Triangular Perspective: Qualitative Study
Source: J Med Internet Res. 2025 Nov 11;27:e72716. doi: 10.2196/72716 (PMC12648130; doi:10.2196/72716)
Supplement: Multimedia Appendix 2 [file jmir_v27i1e72716_app2.docx]

**Focus Group Interview Guide**

This core interview protocol was designed to capture older adults' experiences regarding their daily function, health and technology use. The questions were tailored to each group's unique perspective: for family members, they were framed in relation to "your family member," while for healthcare professionals, they were framed to draw upon their professional experience with "the older adults you work with."

Introduced Question: What is your most enjoyable activity in your free time?

Grand Opening Question: When you think of the moment you got up this morning, what were the first thoughts that came to mind related to your daily activities?

Key Question #1: Reflecting on your typical daily routine, from morning to night, describe points in the day or specific situations where you find you need assistance?

Key Question #2: Think about the activities you want to do versus those you need to do. Describe your energy levels in both types of activities. Please give examples of activities where time seems to fly by, and others where it seems to drag on?

Key Question #3: When you face a task that is difficult for you, what resources or strategies help you complete it? And on the other hand, how do you typically feel or react when you're unable to do something you expect yourself? How frequently would you say this occur?

Transition Question: Now, let's shift our focus. When you hear the phrase "the COVID-19 pandemic," what thoughts or images first come to mind?

Key Question #4: In what ways did the pandemic impact your daily routines and activities compared to the time before it began?

Key Question #5: Reflecting on that period, did your sense of purpose or motivation for starting the day change? If so, can you tell me more about that?

Transition Question: Let's talk about technology. What kinds of devices, such as smartphones, tablets, or computers, do you use in your daily life? For what purposes, and how often? What challenges, if any, do you face when using technology?

Key Question #6: During the pandemic, describe your relationship with technology? For example, did you use it more or less, or for different reasons? In what ways did technology help—or perhaps hinder—your ability to engage in daily activities? Describe times in which you felt frustrated or empowered by technology during that period?

Key Question #7: Thinking back, describe whether there was any technology you wish you had access to then? What would have enabled you to use technology more effectively? Looking forward, what would you like to be able to do with technology that you currently can't?

Concluding Question: Of all the things we've discussed today, what would you say is the single biggest concern or challenge you currently face in your daily life?

If there are other subjects we didn’t discuss relating to your daily routines and function and technology use, please address them.
